# Integrative bioinformatics analysis of lipid metabolism-related genes and immune infiltration in endometriosis

**DOI:** 10.1097/MD.0000000000042816

**Published:** 2025-06-13

**Authors:** Xiaofeng Ye, Xiaoxia Song, Sihang Zhou, Guoqing Chen, Liping Wang

**Affiliations:** a Reproductive Medicine Centre, The First Affiliated Hospital of Shenzhen University, Shenzhen Second People’s Hospital, Guangdong Key Laboratory for Biomedical Measurements and Ultrasound Imaging, National-Regional Key Technology Engineering Laboratory for Medical Ultrasound, School of Biomedical Engineering, Shenzhen University Medical School, Shenzhen, China; b Reproductive Medicine Centre, The First Affiliated Hospital of Shenzhen University, Shenzhen Second People’s Hospital, Shenzhen, China; c Health Science Center, Shenzhen University, Shenzhen, China.

**Keywords:** bioinformatics, endometriosis, eutopic endometrium, immune infiltration, lipid metabolism, midsecretory phase

## Abstract

Endometriosis is referred to as a “benign cancer,” severely impacting women’s reproductive health. However, the pathogenesis of endometriosis remains unclear. This study aims to investigate the potential roles of lipid metabolism-related genes (LMRGs) and immune infiltration in the diagnosis and pathogenesis of endometriosis. Four microarray datasets (GSE6364, GSE51981, GSE153740, and GSE232713) with eutopic endometrium samples of the midsecretory phase were downloaded from the Gene Expression Omnibus database. Seven hundred forty LMRGs were obtained from the Reactome database. The GSE6364 and GSE51981 datasets were merged and differentially expressed genes (DEGs) were identified between endometriosis patients and normal controls. Lipid metabolism-related DEGs were detected by intersecting the DEGs and LMRGs. Functional enrichment analysis, protein–protein interaction analysis, and receiver operating characteristic analysis of lipid metabolism-related DEGs were performed. Additionally, immune cell infiltration was compared between endometriosis patients and normal controls, and associations with lipid metabolism-related DEGs were assessed. Fifty-eight lipid metabolism-related DEGs were identified in endometriosis patients compared with normal controls, which enriched in glycerolipids, fatty acyls, sphingolipids, glycerophospholipids, and sterol lipids metabolism, especially steroid hormone metabolism and arachidonic acid metabolism. Additionally, 11 core genes were identified, with *HMGCR* and *CYP27A1* validated as potential markers for diagnosing endometriosis and assessing its severity, respectively. Immune infiltration analysis revealed that fibroblasts and B lineage cells were predominantly abnormal in the endometrium of the midsecretory phase in endometriosis. Correlation analysis suggested that core genes were closely related to immune cells. In conclusion, *HMGCR* and *CYP27A1* were identified as potential markers for endometriosis and its severity, respectively. Fibroblasts and B lineage cells may significantly contribute to the reduced endometrial receptivity observed in endometriosis. These findings provide new insights into the diagnostic and pathogenic roles of LMRGs in endometriosis and highlight their implications for infertility and pregnancy complications related to endometriosis.

## 1. Introduction

Endometriosis is a complicated gynecological condition defined by the presence of endometrial tissue outside the uterus, typically in the pelvis.^[1]^ Infertility and chronic pain are the common clinical symptoms of endometriosis. Up to 50% of women with infertility and approximately 10% of reproductive-aged women are affected by endometriosis, which has a significant impact on women’s reproductive health and quality of life.^[2]^

Recently, there has been extensive discussion regarding the detrimental impact of endometriosis on fertility. Endometriosis significantly increases the risk of infertility and obstetric complications such as miscarriage and small-for-gestational-age infants.^[3,4]^ These issues are primarily attributed to decreased endometrial receptivity in women with endometriosis.^[5,6]^ Proposed mechanisms for reduced endometrial receptivity include the dysregulation of genes related to uterine receptivity, alterations in immune cell distribution within the endometrium, and epigenetic modifications.^[7–9]^ However, no consensus has been reached on the precise mechanism.

Despite being a benign condition, endometriosis exhibits rapid growth of endometrial cells and clinical features such as recurrence and distant metastasis, which are strikingly similar to cancer.^[10,11]^ This resemblance raises the question of whether the pathogenic mechanisms common in cancer also apply to endometriosis. Notably, lipid metabolism reprogramming, a recently recognized hallmark of malignancy, plays a critical role in cancer progression.^[12]^ It functions as a signaling molecule, energy source, and local immune cell infiltration regulator.^[12,13]^

Thus, our study aims to clarify the role of lipid metabolism-related genes (LMRGs) in endometriosis. We employed bioinformatics approaches to identify differentially expressed genes (DEGs) and lipid metabolism-related DEGs from eutopic endometrium samples collected during the midsecretory phase in patients with endometriosis and normal controls. Furthermore, we investigated the functional enrichment of lipid metabolism-related DEGs, delineated key genes, and assessed the infiltration of immune cells within these samples. This research is crucial for understanding how LMRGs contribute to the observed fertility reduction in endometriosis patients.

## 2. Methods

### 2.1. Datasets

Four datasets containing samples of eutopic endometrial tissue collected during the midsecretory phase, including both normal controls and endometriosis patients—GSE6364, GSE51981, GSE153740, and GSE232713—were obtained from the Gene Expression Omnibus database (https://www.ncbi.nlm.nih.gov/geo/). Detailed information about these datasets can be found in Supplementary Table 1, Supplemental Digital Content, https://links.lww.com/MD/P178.

To identify DEGs between the normal control group and the endometriosis group, we utilized the GSE6364 and GSE51981 datasets. For validation of our findings, we employed the GSE153740 and GSE232713 datasets. LMRGs were sourced from the Reactome database, resulting in the identification of a total of 740 relevant genes.^[14]^ These genes are detailed in Supplementary Table 2, Supplemental Digital Content, https://links.lww.com/MD/P179.

### 2.2. Identification of lipid metabolism-related DEGs

We used the R software (v 4.3.2; R Core Team, Vienna, Austria) package called “insilicomerging” to merge the datasets GSE6364 and GSE51981.^[15]^ Next, we applied a method developed by Johnson et al^[16]^ to eliminate any batch effects that could potentially skew our results. Subsequently, we reduced the complexity of our data using the “umap” package in R (v 4.3.2), which allows us to visualize high-dimensional data in a lower-dimensional space.

To identify DEGs between the endometriosis patients and the control group, we utilized the “limma” package in R (v 4.3.2). The statistical significance cutoff criteria were established with a |log fold change| (log FC) > 0.585 and *P* value < .05. Then, the DEGs and LMRGs were compared using online Venn software (http://www.xiantaozi.com) to determine the lipid metabolism-related DEGs.

### 2.3. Functional enrichment analysis

To clarify the functions of lipid metabolism-related DEGs in endometriosis, we conducted Kyoto Encyclopedia of Genes and Genomes (KEGG) and Gene Ontology (GO) analyses. These analyses were performed using the Database for Annotation, Visualization, and Integrated Discovery (DAVID) at the following website: http://david.ncifcrf.gov/home.jsp. The results of the pathway enrichment analysis were presented using an online platform available at https://www.bioinformatics.com.cn.

### 2.4. PPI network analysis and core genes identify

To create a protein–protein interaction (PPI) network, we used the Search Tool for the Retrieval of Interacting Genes (STRING) database. This database allows us to find and analyze how different proteins interact with each other. Once we gathered the interaction data, we visualized the network using Cytoscape software, which helped us create a graphical representation of these interactions.

After constructing the network, we focused on identifying the core genes that play crucial roles within this network. We used a specific measure called “betweenness centrality” to determine the importance of each gene in the network. In our analysis, we defined core genes as those with a betweenness centrality score >100, which indicates that these genes are key connectors in the network and potentially influence many other genes. This approach helped us pinpoint the most significant proteins involved in the interactions related to our study.

### 2.5. ROC analysis

The predictive ability of the identified core genes for diagnosing endometriosis was assessed in GSE153740 and GSE232713 by calculating the area under the receiver operating characteristic (ROC) curve, utilizing the OmicStudio tools at https://www.omicstudio.cn/tool. Similarly, the predictive capacity of the identified core genes for assessing the severity of endometriosis was examined in GSE51981 utilizing the same methodology.

### 2.6. Immune cell infiltration analysis

Based on the gene expression profiles we collected, we analyzed the amount of different immune cell types present in each sample using a tool called microenvironment cell populations-counter, which is part of an R software (v 4.3.2) package.^[17]^ Subsequently, the differences in immune cell populations between the endometriosis and control groups were illustrated with the boxplot function from the R (v 4.3.2) package.

### 2.7. Correlation analysis between immune cells and core genes

The correlation between immune cells and the 11 core genes was investigated using Pearson correlation analysis in OmicStudio Tools at https://www.omicstudio.cn/tool, and a correlation plot was generated to visually illustrate the results.

## 3. Results

### 3.1. Identification of lipid metabolism-related DEGs

**Figure 1.**
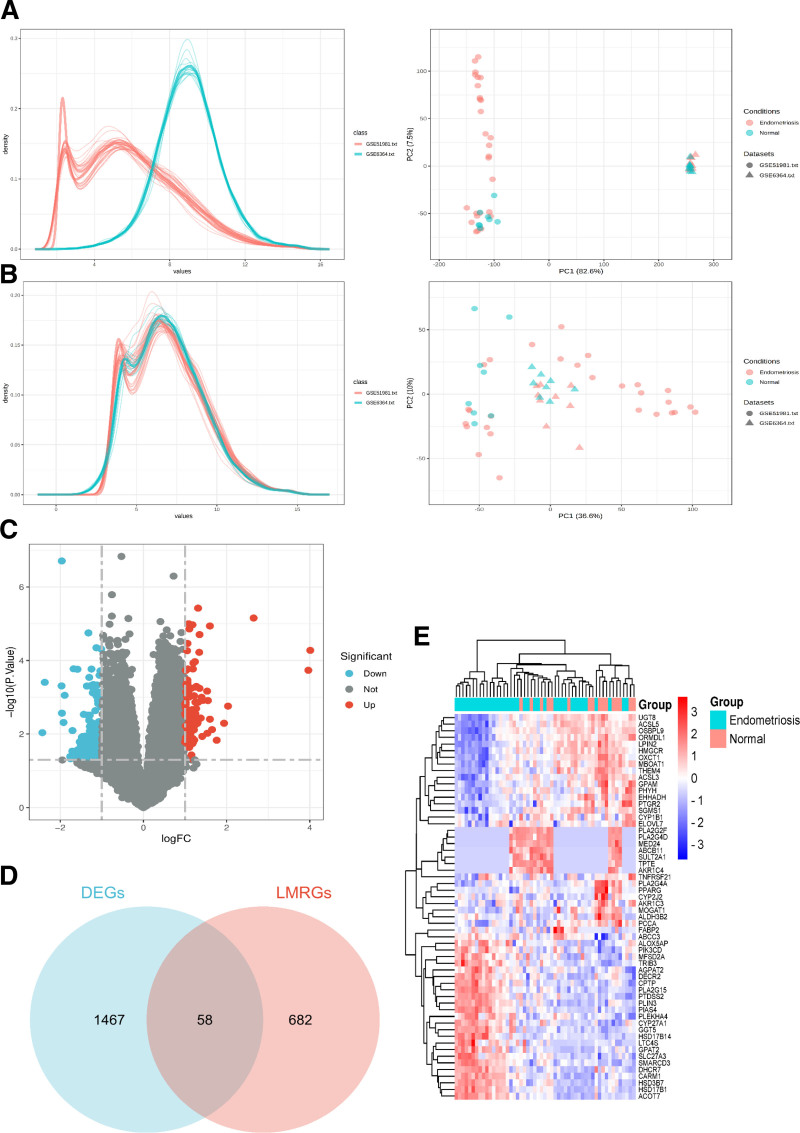
Identification of lipid metabolism-related DEGs. (A) Density distribution and PCA before adjusting for batch effects. (B) Density distribution and PCA after adjusting for batch effects. (C) Volcano plot of DEGs between women with endometriosis and normal controls, red dots represent upregulated genes, blue dots represent downregulated genes, and gray dots represent normal genes. (D) Venn diagram of 1525 DEGs and 740 LMRGs. (E) Heatmap of the 58 lipid metabolism-related DEGs. DEGs = differentially expressed genes, LMRGs = lipid metabolism-related genes, PCA = principal component analysis

The Gene Expression Omnibus datasets GSE6364 and GSE51981 included midsecretory phase eutopic endometrial samples from 16 normal women and 37 women with endometriosis. Upon merging the datasets and removing batch effects, the data distribution among them appeared consistent (Fig. 1A, B).

In the combat dataset, 1525 DEGs were identified between endometriosis and normal women (Supplementary Table 3, Supplemental Digital Content, https://links.lww.com/MD/P180). The volcano plots illustrating these DEGs can be seen in Figure 1C. Subsequent Venn analysis confirmed 58 lipid metabolism-related DEGs among the DEGs and LMRGs (Fig. 1D, Supplementary Table 4, Supplemental Digital Content, https://links.lww.com/MD/P181). The heatmap of these 58 lipid metabolism-related DEGs is shown in Figure 1E.

### 3.2. Enrichment analysis of lipid metabolism-related DEGs

GO enrichment and KEGG pathway analyses revealed the functional roles associated with the 58 lipid metabolism-related DEGs in endometriosis. These DEGs were found to be predominantly associated with various cellular components, biological processes, and molecular functions of lipid metabolism. Specifically, they were linked to 62 biological process terms (bile acid biosynthetic process, fatty acid metabolic process, long-chain fatty acid transport, etc), 15 cellular component terms (endoplasmic reticulum membrane, endoplasmic reticulum, lipid particle, integral component of membrane, etc), and 51 molecular function terms (17-beta-hydroxysteroid dehydrogenase [NAD+] activity, testosterone 17-beta-dehydrogenase [NADP+] activity, dihydrotestosterone 17-beta-dehydrogenase activity, etc).

**Figure 2.**
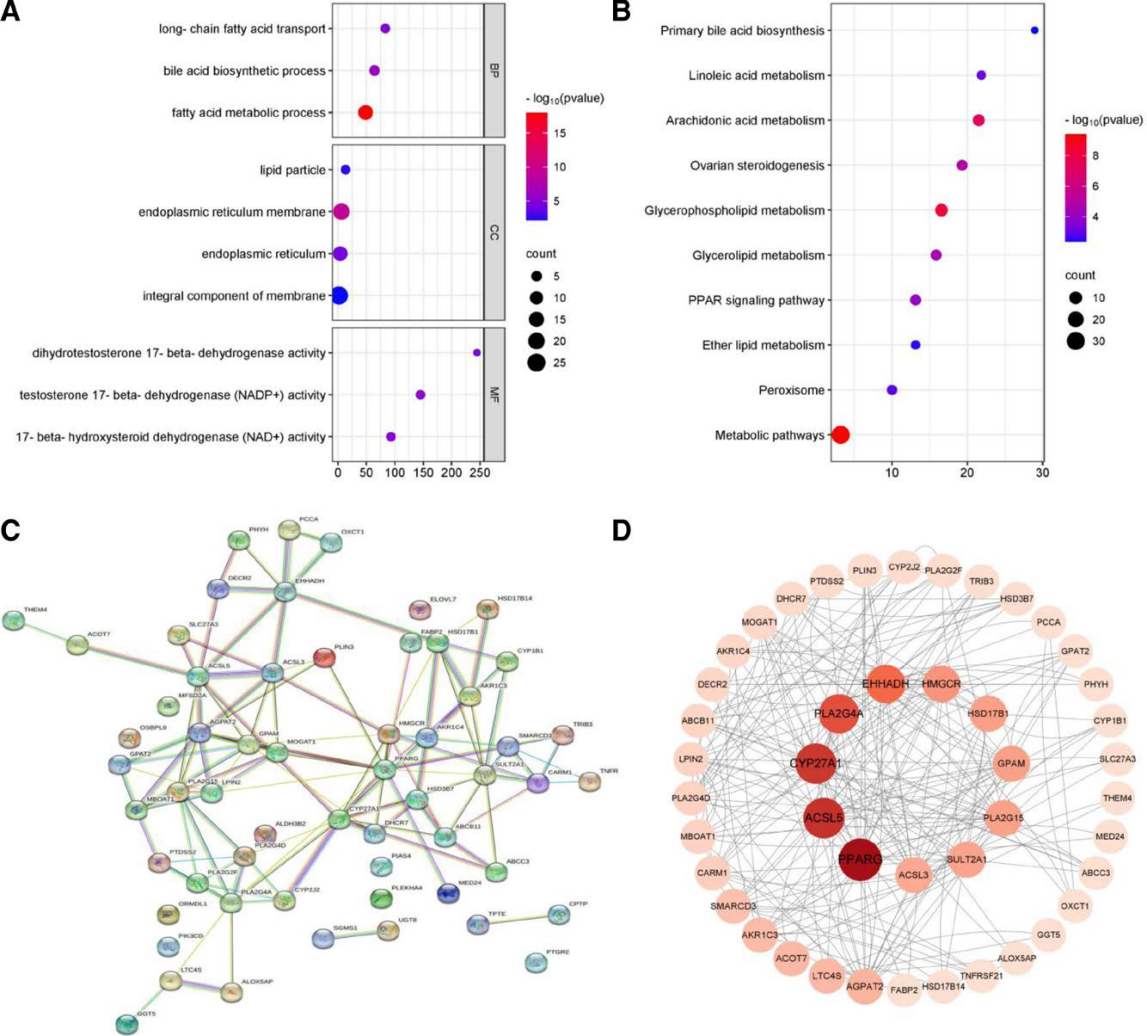
Functional enrichment analysis and PPI network. (A) GO functional analysis of lipid metabolism-related DEGs. (B) KEGG pathway analysis of lipid metabolism-related DEGs. (C) PPI analysis of lipid metabolism-related DEGs using STRING. (D) PPI analysis of lipid metabolism-related DEGs using Cytoscape to identify 11 core genes. DEGs = differentially expressed genes, GO = gene ontology, KEGG = Kyoto Encyclopedia of Genes and Genomes, PPI = protein–protein interaction, STRING = Search Tool for the Retrieval of Interacting Genes.

Furthermore, the KEGG analysis highlighted several altered signaling pathways in endometriosis-related to lipid metabolism, encompassing metabolic pathways, arachidonic acid metabolism, glycerophospholipid metabolism, ovarian steroidogenesis, glycerolipid metabolism, linoleic acid metabolism, peroxisome proliferator-activated receptor signaling pathway, peroxisome, ether lipid metabolism, primary bile acid biosynthesis, and others. Detailed results can be found in Figure 2A, B, as well as Supplementary Table 5, Supplemental Digital Content, https://links.lww.com/MD/P182 and Supplementary Table 6, Supplemental Digital Content, https://links.lww.com/MD/P183.

Overall, upon combining all enriched pathways, the steroid hormone and arachidonic acid pathways emerged as the 2 most significantly enriched pathways.

### 3.3. PPI network and core gene identification

The PPI network analysis of lipid metabolism-related DEGs revealed the presence of 58 nodes and 116 edges (Fig. 2C). Subsequently, the network was imported into Cytoscape to pinpoint core genes within it. Utilizing the BETWEENNESS algorithm in CytoNCA, 11 core genes, including *GPAM, PPARG, CYP27A1, ACSL5, PLA2G4A, EHHADH, HSD17B1, HMGCR, PLA2G15, SULT2A1,* and *ACSL3*, surpassing the set threshold were successfully identified (Fig. 2D, Supplementary Table 7, Supplemental Digital Content, https://links.lww.com/MD/P184).

### 3.4. ROC analysis

**Figure 3.**
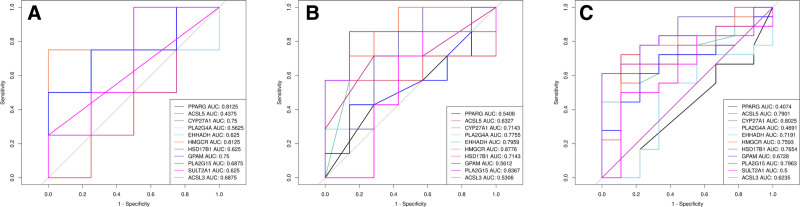
ROC analysis. (A) Predictive ability of the 11 core genes for diagnosing endometriosis in patients from GSE153740. (B) Predictive ability of the 11 core genes for diagnosing endometriosis in patients from GSE232713. (C) Predictive ability of the 11 core genes for assessing endometriosis severity in patients from GSE51981. ROC = receiver operating characteristic curve.

ROC analysis was conducted on the 11 core genes to evaluate their potential diagnostic utility for endometriosis in the GSE153740 and GSE232713 datasets. The area under the curve values for *PPARG*, *ACSL5*, *CYP27A1*, *PLA2G4A*, *EHHADH*, *HMGCR*, *HSD17B1*, *GPAM*, *PLA2G15*, *SULT2A1*, and *ACSL3* were 0.812, 0.438, 0.75, 0.563, 0.625, 0.813, 0.625, 0.75, 0.688, 0.625, and 0.688 in GSE153740 dataset and 0.541, 0.633, 0.714, 0.776, 0.796, 0.878, 0.714, 0.561, 0.837, NA, and 0.531 in GSE232713 dataset (Fig. 3A, B). Additionally, ROC analysis was carried out on the same 11 core genes to assess their potential diagnostic accuracy for endometriosis severity in the GSE51981 dataset. The area under the curve values for *PPARG, ACSL5, CYP27A1, PLA2G4A, EHHADH, HMGCR, HSD17B1, GPAM, PLA2G15, SULT2A1*, and *ACSL3* were 0.407, 0.79, 0.803, 0.469, 0.719, 0.759, 0.765, 0.673, 0.796, 0.5, and 0.624, respectively (Fig. 3C). Overall, *HMGCR* and *CYP27A1* seem to be promising biomarkers for the identification of endometriosis and the assessment of its severity, respectively.

### 3.5. Immune cell infiltration analysis

**Figure 4.**
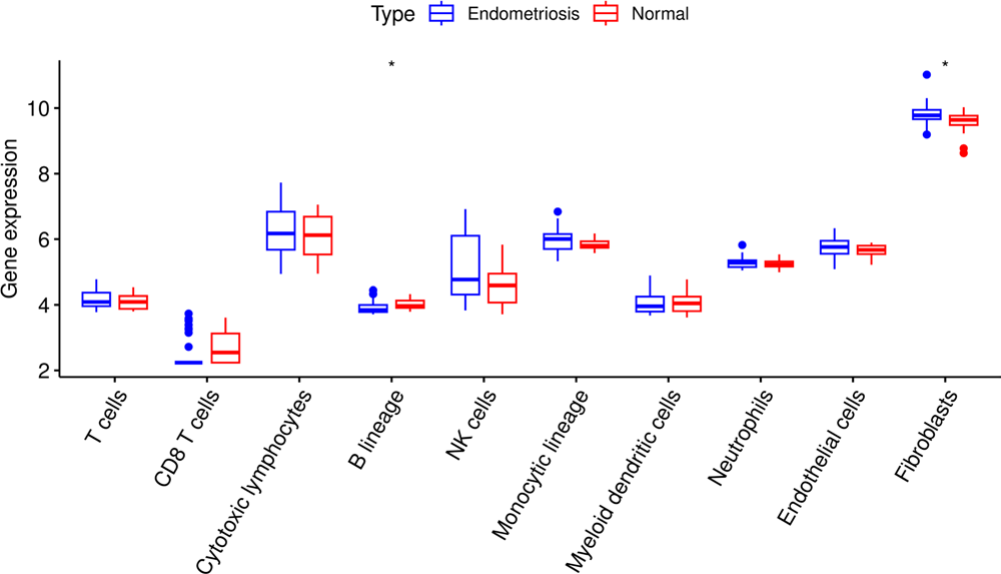
Boxplot comparing immune infiltration levels between women with endometriosis and normal controls.

The immune cell infiltration scores were computed with the microenvironment cell populations -counter method for each sample, utilizing the combat gene expression profile. The results revealed significant differences in the number of B lineage cells and fibroblasts between women with endometriosis and normal controls (Fig. 4). However, no meaningful differences were found between the groups for CD8 T cells, T cells, NK cells, cytotoxic lymphocytes, myeloid dendritic cells, monocytic lineage, neutrophils, and endothelial cells.

### 3.6. Correlation analysis between immune cells and core genes

**Figure 5.**
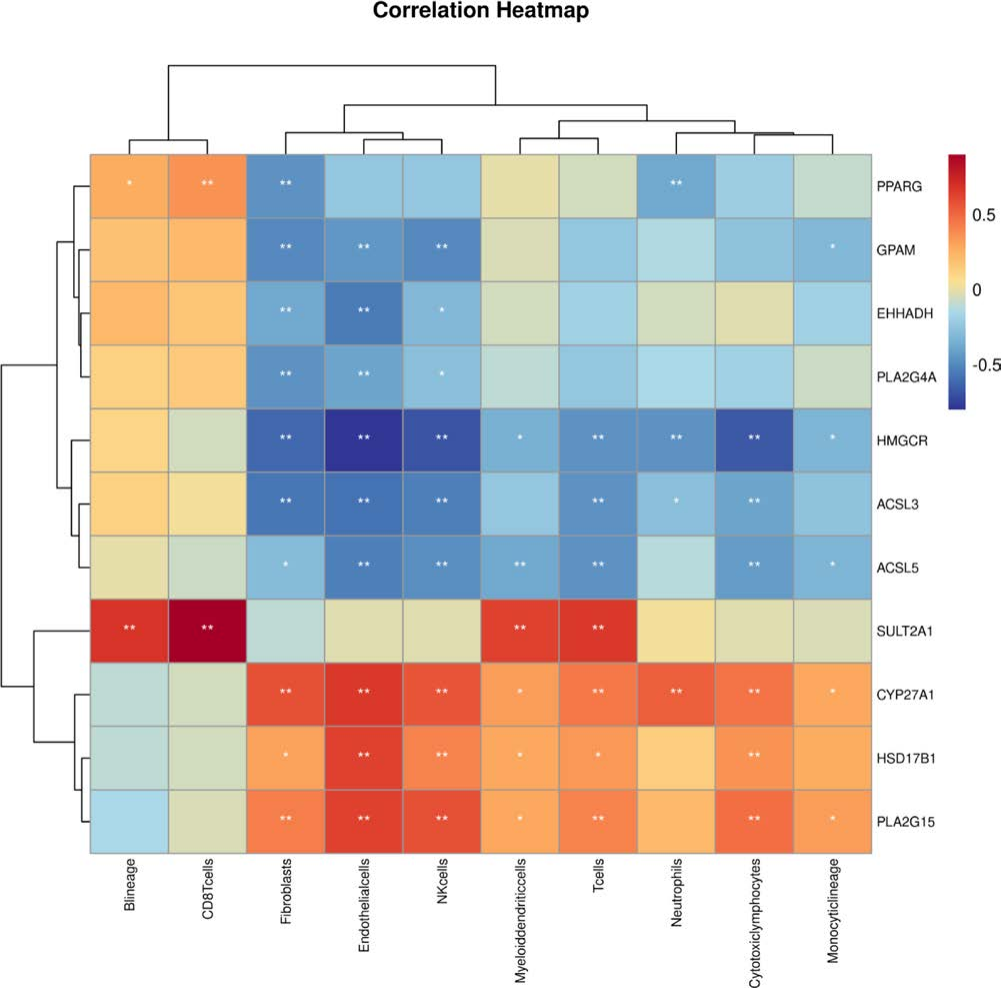
Correlation analysis between the immune cells and the 11 core genes. *P < .05, **P < .01.

To enhance our understanding of the role of lipid metabolism-related DEGs in endometriosis, the interaction between immune cells and 11 core genes was explored. As depicted in Figures 5, 10 core genes, except for *SULT2A1*, were linked to fibroblasts. Furthermore, *PPARG* and *SULT2A1* showed associations with B lineage cells.

## 4. Discussion

Sampson theory regarding retrograde menstruation is largely accepted as the principal cause of endometriosis.^[1,18]^ Even though 90% of women undergo retrograde menstruation, only 15% go on to develop endometriosis. Some studies have advised that this discrepancy may be attributed to abnormal functioning of the eutopic endometrium.^[1,19]^ This aligns with research indicating that dysfunctional eutopic endometrium is linked to reduced fertility in individuals with endometriosis, underscoring the crucial role of eutopic endometrium in the development of endometriosis.^[7,20]^ In recent years, metabolic factors have been increasingly recognized as contributors to endometriosis, with notable abnormalities in lipid metabolism observed in patients suffering from the condition.^[21]^ Lipid metabolism has been associated with cell invasion and abnormal immune cell infiltration and is closely tied to endometrial receptivity.^[13,22]^ Consequently, the present study delved into the potential impact of LMRGs on endometriosis.

By analyzing 2 transcriptomic datasets from the midsecretory phase of the endometrium, 58 dysregulated LMRGs in endometriosis were recognized, which are involved in various metabolic processes, particularly with significant enrichment in the steroid hormone and arachidonic acid pathways. Additionally, 11 core genes have been pinpointed, with *HMGCR* and *CYP27A1* validated as potential biomarkers for the identification and severity assessment of endometriosis, respectively. Analysis of immune cell infiltration revealed an abnormal predominance of B lineage cells and fibroblasts in the midsecretory phase of eutopic endometrium in endometriosis. Moreover, correlation analysis indicated a close relationship between the immune cells and core genes.

The human endometrium undergoes cyclical changes in reaction to fluctuations in estrogen and progesterone levels, transitioning through the proliferative, secretory, and menstrual phases.^[23]^ Lipid metabolism in endometrial cells is also hormonally regulated, exhibiting distinct patterns throughout these phases. During the proliferative phase, endometrial epithelial cells exhibit low lipid content, primarily synthesizing phospholipids that are essential for plasma membrane composition, which reflects cellular proliferation. As the endometrium advances into the secretory phase under progesterone influence, lipid accumulation within the endometrial epithelium increases, indicating heightened metabolic activity.^[24–26]^ This lipid buildup serves as an energy source for the developing embryo, however, excessive lipid accumulation during the late secretory phase can lead to epithelial dysfunction, resulting in inflammation, cell necrosis, and ultimately menstruation.^[25,27]^ Thus, the dynamic lipid metabolism in the endometrial epithelium is crucial for both conception and menstruation.

Our study focused on the midsecretory phase of eutopic endometrium, a period characterized by heightened lipid metabolism, providing insights into the impact of lipid metabolites on epithelial function.^[28]^ Our analysis revealed significant differences in LMRGs in endometriosis, with enrichment observed in phospholipid, sphingolipid, and fatty acid metabolism, particularly in steroid hormone and arachidonic acid pathways. Perturbations in steroid receptor expression and function, such as decreased ERα and increased ERβ expression, as well as reduced PR expression and insensitivity leading to progesterone resistance, are well documented in endometriosis.^[1,29]^ Changes in the endometrium driven by steroid hormones may be vital in the progression of endometriosis. For instance, the estrogen/H19/ACTA2 axis regulates the migration and invasion of stromal cells in eutopic endometrium.^[30]^ Arachidonic acid, a common fatty acid derived from polyunsaturated fats in the diet, is involved in the synthesis of prostaglandins, lipotoxin A4, and the cannabinoid system, thromboxanes, and leukotrienes, contributing to chronic inflammation, dysmenorrhea in endometriosis, and abnormalities in embryo implantation by modulating angiogenesis and immunity.^[31–33]^ Overall, our study emphasizes the significance of lipid metabolism, particularly in steroid hormone and arachidonic acid pathways, in contributing to endometriosis development.

Our study suggests that multiple genes could be crucial in the advancement of endometriosis. Among these genes, *CYP27A1*, which codes for 27-hydroxylase, a member of the cytochrome P450 superfamily, facilitates the conversion of cholesterol into 27-hydroxycholesterol (27-HC). *CYP27A1* is implicated in numerous biological processes, including cholesterol metabolism, steroid synthesis, and metabolism, bile acid biosynthesis, and the biological function of vitamin D3.^[34,35]^ Currently, there is no literature discussing the involvement of *CYP27A1* in endometriosis. However, recent research has highlighted its potential relevance in obstetrics and gynecology. Studies have linked 27-HC to placental trophoblast fusion disorders such as pre-eclampsia.^[36,37]^ Exploring the potential role of *CYP27A1* in endometriosis is a promising area for further research.

*HMGCR* is an essential part of the cholesterol synthesis pathway, playing a key role in the mevalonate pathway. Statins, which are potent competitive inhibitors of *HMGCR*, are currently being studied for their effects on endometriosis.^[38,39]^ Animal research has shown that statins can diminish the size of endometriotic lesions by exerting anti-inflammatory effects, inhibiting cell proliferation, promoting cell death, and reducing the production of vascular endothelial growth factor.^[40,41]^ A human study has also demonstrated that statins can alleviate postoperative pain in individuals with endometriosis, indirectly indicating the significance of *HMGCR* in the development of the condition.^[42]^ Our findings offer direct evidence of *HMGCR*’s involvement in the pathogenesis of endometriosis, yet the precise mechanism warrants additional investigation.

The link between *PPARG* and endometriosis has been established in multiple studies, revealing discrepancies in *PPARG* gene polymorphisms and expression levels between endometriosis patients and normal controls.^[43]^ Animal experiments have demonstrated that *PPARG* agonists can attenuate inflammation, invasion, angiogenesis, and adhesion of endometrial lesions while promoting apoptosis.^[44,45]^ *HSD17B1* is essential for steroid hormone metabolism, particularly in estrogen biosynthesis and regulation.^[46]^ It catalyzes the conversion of estrone to the more potent estrogen, estradiol, leading to increased local estrogen levels in ectopic endometrial tissue, stimulating cell proliferation, and contributing to the pathogenesis of endometriosis.^[47]^ Our study suggests that abnormal *HSD17B1* expression in eutopic endometrial tissue may be associated with reduced endometrial receptivity. There has been limited research on the roles of *ACSL5*, *PLA2G4A*, *EHHADH*, *GPAM*, *PLA2G15*, *SULT2A1*, and *ACSL3* in endometriosis, highlighting the necessity for further investigation to elucidate their contributions to its pathogenesis.

Our findings indicated that fibroblasts and B cells exhibit the most notable variances between endometriosis and healthy individuals during the midsecretory phase of eutopic endometrium. Research has highlighted the abnormal quantity and function of fibroblasts in eutopic and ectopic endometrium in endometriosis, with more severe fibrosis observed in ectopic endometrium.^[48,49]^ Fibrosis plays a crucial role in the pain and infertility associated with endometriosis. In endometriosis, dormant fibroblasts transform into activated myofibroblasts, aided by chronic inflammation and other stimuli, allowing them to proliferate, adhere, and migrate in vitro.^[48]^ Pseudotime analysis of fibroblasts through single-cell sequencing further bolsters the idea that ectopic endometrium originates from eutopic endometrium.^[49]^ Furthermore, fibroblasts in eutopic endometrium are associated with progesterone resistance, leading to impaired decidualization and abnormal embryo implantation.^[50]^

B cells constitute a limited population within the endometrium and are responsible for humoral immune responses.^[51]^ Studies have revealed perturbed numbers and functions of B cells in endometriosis patients.^[52]^ A Mendelian study indicated a significant link between B cell proportions and the heightened risk of endometriosis.^[53]^ Animal experiments have demonstrated the efficacy of B cell inhibitors in reducing endometriotic implant size, highlighting the critical role of B cells in this condition.^[54]^ B cells are implicated in the pathogenesis of endometriosis by producing autoantibodies, which can bind to the endometrium, embryos, and sperm, leading to infertility and pregnancy complications in endometriosis subjects.^[51,52]^

The mechanisms underlying the abnormal presence of fibroblasts and B cells in the eutopic endometrium of endometriosis patients remain unclear. Our research has unveiled a potential association between lipid metabolism genes and the dysregulation of fibroblasts and B cells, providing insight into a possible underlying mechanism for these anomalies.

## 5. Conclusion

Our study is the pioneering investigation into the involvement of LMRGs in developing endometriosis, specifically targeting the midsecretory phase crucial for embryo implantation. This research not only provides evidence regarding the possible origins of eutopic endometrium in endometriosis but also offers vital insights into understanding infertility and pregnancy-related complications caused by this endometriosis.

Our study reveals notable dysregulation of LMRGs and pathways in endometriosis patients compared with normal controls, as well as distinct immune cell infiltration patterns in the midsecretory phase of eutopic endometrium in endometriosis. While our comprehensive bioinformatics analyses provide valuable insights, further in vitro and in vivo studies are crucial to confirm the roles of LMRGs in endometriosis and to deepen our understanding of its underlying mechanisms.

## Acknowledgments

The authors extend their gratitude to Burney RO and his colleagues for providing the GSE6364 dataset, Tamaresis JS and his colleagues for providing the GSE51981 dataset, Bane K and his colleagues for providing the GSE153740 dataset, and Li X and his colleagues for providing the GSE232713 dataset.

## Author contributions

**Formal analysis:** Xiaofeng Ye, Xiaoxia Song.

**Software:** Xiaofeng Ye, Xiaoxia Song.

**Visualization:** Xiaofeng Ye.

**Writing – original draft:** Xiaofeng Ye.

**Writing – review & editing:** Xiaoxia Song, Sihang Zhou, Guoqing Chen, Liping Wang.

**Data curation:** Sihang Zhou, Guoqing Chen.

**Conceptualization:** Liping Wang.

**Funding acquisition:** Liping Wang.

## Supplementary Material


